# Evaluation of Diagnostic Yield in Fetal Whole-Exome Sequencing: A Report on 45 Consecutive Families

**DOI:** 10.3389/fgene.2019.00425

**Published:** 2019-06-25

**Authors:** Lior Greenbaum, Ben Pode-Shakked, Shlomit Eisenberg-Barzilai, Michal Dicastro-Keidar, Anat Bar-Ziv, Nurit Goldstein, Haike Reznik-Wolf, Hana Poran, Amihai Rigbi, Ortal Barel, Aida M. Bertoli-Avella, Peter Bauer, Miriam Regev, Annick Raas-Rothschild, Elon Pras, Michal Berkenstadt

**Affiliations:** ^1^The Danek Gertner Institute of Human Genetics, Sheba Medical Center, Tel Hashomer, Israel; ^2^The Joseph Sagol Neuroscience Center, Sheba Medical Center, Tel Hashomer, Israel; ^3^Sackler Faculty of Medicine, Tel Aviv University, Tel Aviv, Israel; ^4^Faculty of Education, Beit Berl College, Kfar Saba, Israel; ^5^Sheba Cancer Research Center, Sheba Medical Center, Tel Hashomer, Israel; ^6^Centogene AG, Rostock, Germany

**Keywords:** whole-exome sequencing (WES), prenatal diagnosis, ultrasound abnormalities, clinical genetics, congenital anomalies

## Abstract

Prenatal ultrasound (US) abnormalities often pose a clinical dilemma and necessitate facilitated investigations in the search of diagnosis. The strategy of pursuing fetal whole-exome sequencing (WES) for pregnancies complicated by abnormal US findings is gaining attention, but the reported diagnostic yield is variable. In this study, we describe a tertiary center’s experience with fetal WES from both terminated and ongoing pregnancies, and examine the clinical factors affecting the diagnostic rate. A total of 45 consecutive families of Jewish descent were included in the analysis, for which clinical fetal WES was performed under either single (fetus only), trio (fetus and parents) or quatro (two fetuses and parents) design. Except one, all families were non-consanguineous. In 41 of the 45 families, WES was sought following abnormal fetal US findings, and 18 of them had positive relevant family history (two or more fetuses with US abnormalities, or single fetus with US abnormalities and an affected parent). The overall diagnostic yield was 28.9% (13/45 families), and 31.7% among families with fetal US abnormalities (13/41). It was significantly higher in families with prenatal US abnormalities and relevant family history (10/18, 55.6%), compared to families with prenatal US abnormal findings and lack of such history (3/23, 13%) (*p* = 0.004). WES yield was relatively high (42.9–60%) among families with involvement of brain, renal or musculoskeletal US findings. Taken together, our results in a real-world setting of genetic counseling demonstrates that fetal WES is especially indicated in families with positive family history, as well as in fetuses with specific types of congenital malformation.

## Introduction

Recent years have seen the growing implementation of next-generation sequencing (NGS) techniques into widespread clinical use, revolutionizing the diagnostic odyssey for many families with monogenic disorders (Yang et al., 2013; Stranneheim and Wedell, 2016). While whole-exome sequencing (WES) and whole-genome sequencing (WGS) are more commonly utilized as a tool for molecular diagnosis of affected pediatric and adult patients (Lee et al., 2014; Taylor et al., 2015; Sawyer et al., 2016), data regarding their utility in routine use at the prenatal setting is still relatively limited (Jelin and Vora, 2018).

Initially, several preliminary reports described the successful use of fetal WES/WGS in single families (Talkowski et al., 2012; Filges et al., 2014). Since then, several groups have reported their experience with exome sequencing in the context of fetuses detected by ultrasound (US) to have structural anomalies (for example, Carss et al., 2014; Drury et al., 2015; Fu et al., 2018; Normand et al., 2018). The widely variable diagnostic rates in literature (ranging from around 6.2% to as high as 80%) may be attributable to differences in inclusion criteria, single (fetus only) versus trio sequencing and sample size (Best et al., 2018). Diagnostic yield was higher in cases of, for instance, parental consanguinity, previous normal karyotype and chromosomal microarray analysis (CMA), fetuses with multiple congenital anomalies or fetal features suggestive of a specific syndrome, related to variants in multiple known causative genes (Vora et al., 2017; Best et al., 2018; Daum et al., 2019). Recently, two large scales studies of fetal WES were published. In the Prenatal Assessment of Genomes and Exomes (PAGE) study, which included exome sequencing in 610 fetuses (and 1202 parental samples) with structural anomalies detected by US, a diagnostic genetic variant was found in 8.5% of the fetuses, and additional 3.9% of fetuses had variant of unknown significance (VUS) with potential clinical usefulness (Lord et al., 2019). In another WES study of fetuses with structural anomalies, diagnostic rate of 10.3% among 234 fetus-parents trios was reported (Petrovski et al., 2019).

We describe herein our experience with clinical fetal WES in 45 consecutive families from either terminated or ongoing pregnancies, and discuss the added value and challenges associated with this diagnostic strategy. The study represents a real-world setting in genetic counseling service, in which mixture of various indications for WES is encountered, and WES is performed for clinical purposes and paid by families.

## Materials and Methods

### Sample Recruitment

In this consecutive series study, we included all families who were consulted at the Danek Gertner Institute of Human Genetics at Sheba Medical Center (SMC) between January 2015 and September 2018, and for whom at least one fetal WES was performed, from a terminated or an ongoing pregnancy. A total of 45 families were retrospectively included in the current study.

All families were of Jewish ancestry, 44 were non-consanguineous and in one family the parents were first degree cousins. Prior to pregnancy or at its first stages, all families were instructed to undertake prenatal genetic carrier screening for prevalent diseases, according to their ethnic origin. All couples received genetic counseling at the SMC genetic institute and a detailed clinical evaluation, including at least one prenatal US examination, and family medical history were obtained. Fetal magnetic resonance imaging (MRI) was carried out in several pregnancies, in addition to US. We included in this study both fetuses with or without abnormal US findings.

WES was performed as a clinical service, paid for directly by the families (for two families, internal funding of the genetic institute was used), and final WES report was issued by the performing lab. Both parents provided written informed consent for the clinical WES, after they had received an explanation regarding the benefits and limitations of the test.

Fetal DNA was extracted following amniocentesis, chorionic villus sampling (CVS) or from fetal material taken during termination of pregnancy (TOP) procedure, and was stored for genetic testing. All TOPs were approved by the institutional committee. Parental DNA was extracted from peripheral blood samples. In addition, prior to WES, CMA was completed in 42 of the families (in at least single fetus) at the SMC cytogenetic laboratory, as a routine clinical service. In all these cases, no cytogenetic findings that explained the phenotype were detected. In the three families with missing CMA, molecular diagnosis was found by WES.

### Whole-Exome Sequencing

Thirty three families performed trio (fetus-mother-father) WES, 7 families quatro (two fetuses-mother-father) WES and in 5 families a singleton WES was sent, depending on availability of fetal DNA from previously terminated pregnancies, as well as financial considerations.

The majority of WES (32 out of 45) were performed at Centogene laboratories^1^. Briefly, commercial capture kits for exome sequencing were used, and sequencing was performed on Ilumina platform, to obtain an average coverage depth of approximately 100X. Bioinformatics analysis included alignments of reads to reference genome, filtering out low quality reads and artifacts, and annotation of variants as described previously (Trujillano et al., 2017). Disease causing variants within the Human Gene Mutation Database (HGMD)^2^, ClinVar^3^ or in CentoMD^4^ as well as variants with a minor allele frequency (MAF) of less than 1% in the Exome Aggregation Consortium (ExAC)^5^ were considered, focusing on exons and flanking intronic bases. The family history and clinical description provided were used to evaluate the identified variants.

Eight of WES were performed at the bioinformatics unit of SMC Cancer Research Center, using similar WES and bioinformatics methodology, and five in CeGaT laboratories^6^. In five cases, the WES raw data was further transferred to an independent bioinformatitian for re-analysis, paid for directly by the family.

Exome data were interpreted according to the American College of Medical Genetics and Genomics (ACMG) guidelines (Richards et al., 2015), and variants were classified as either pathogenic, likely pathogenic, uncertain significance, likely benign or benign.

### Molecular Diagnosis

The final molecular diagnosis to each family was given by the SMC genetic institute team, based on the WES report variant classification and consistency between suggested genes and phenotype. A family was considered as molecularly diagnosed when pathogenic or likely pathogenic variants were found in genes that were pertinent to the clinical phenotype and in line with the suspected inheritance pattern. When variants were classified as VUS in the original WES report, re-evaluation was performed based on clinical considerations, bioinformatics and segregation analysis in additional family members. Team decision was made accordingly.

#### Data Analysis

Families were divided into three groups:

(1) Abnormal prenatal US findings and positive relevant family history: defined as families with two or more fetuses with abnormal prenatal US findings (in current and previous pregnancies), or families with a single fetus with US abnormalities and an affected parent, mother or father (with a resembling phenotype, or potentially manifesting carrier mother). This relates to options of autosomal recessive, autosomal dominant and X-linked models of inheritance, and even of gonadal mosaicism.
(2) Abnormal prenatal US findings without relevant family history: referring to families with a single fetus affected by abnormal US findings, but without any previous fetuses or family members (including adult siblings) with abnormal prenatal US or a medical history relevant to the findings.
(3) Fetuses without any US abnormalities (with or without first degree relative affected by a severe genetic disorder that was excluded in the fetus prior to WES, by Sanger sequencing).

For the diagnostic rate calculations, each family was counted as a single case. To evaluate the effect of fetal US abnormalities type on WES referral and diagnostic yield, we classified the families into five phenotypic groups (based on findings in at least one fetus): brain, renal, cardiac or musculoskeletal system abnormalities and increased nuchal translucency (NT)/edema/hydrops signs. Part of the families matched to more than one group, while others to none.

For statistical analysis, we used *T*-test, Pearson chi-square or Fisher’s exact test (two sided), as appropriate. *P*-value < 0.05 was considered statistically significant.

## Results

Characteristics of 45 fetal WES families included in the presented study and type of WES (single/trio/qautro) are presented in Table 1. In 41 of the 45 families, WES was performed due to a wide range of prenatal US abnormalities. Average maternal age at the pregnancy of the index fetus was 33.5 ± 4.2 years (range, 23–43 years). The pregnant women were at an average gestational age of 23 ± 7.5 weeks (range, 12.5–36 weeks) at first genetic counseling in our clinic (for the index fetus). For 23 families, WES was sent after TOP (51.1%), and in 22 families during an ongoing pregnancy (48.9%). Turnaround time from WES submission to results was 15 to 20 working days for ongoing pregnancies, and up to 2 months following TOP.

**Table 1:** Characteristics of 45 fetal WES families included in the presented study, type of WES and rate of molecular diagnosis.

Families included	Total number	Maternal age at pregnancy (years mean, SD)	Fetal WES performed during ongoing pregnancy (N,%)	Single/Trio/Quatro WES	Families with molecular diagnosis (N,%)
Overall	45	33.5 (4.2)	22 (48.9%)	5/33/7	13 (28.9%)

(1) Abnormal prenatal US findings and positive relevant family history	18	32.4 (4)	4 (22.2%)	2/9/7	10 (55.6%)
- Two or more fetuses with abnormal prenatal US findings (^∗^)	15	32.1 (4.1)	2 (13.3%)	1/7/7	8 (53.3%)
- Single fetus with US abnormalities and an affected parent	3	34.3 (3.8)	2 (66.7%)	1/2/0	2 (66.7%)

(2) Single fetus with abnormal prenatal US findings and lack of relevant family history	23	34.3 (4.5)	14 (60.9%)	1/22/0	3 (13%)

(3) Fetuses without any US abnormalities	4	33.8 (2.4)	4 (100%)	2/2/0	0

*In families with two or more affected fetuses, maternal age at pregnancy relates to pregnancy of the later fetus included in the WES.*

*^∗^In one family, the father and two fetuses were affected.*

Overall, 13 of the 45 families met criteria for a molecular diagnosis, reaching diagnostic yield of 28.9% (Table 1). Among families with US abnormalities, the yield was 13/41 (31.7%). We detected three *de novo* heterozygous causative variants, one autosomal dominant causative variant inherited from the mother, eight autosomal recessive cases (three compound heterozygous and five with homozygous variants) and one X-linked causative variant. Osteogenesis imperfecta due to *de novo* causative variant in the *COL1A2* gene was found in two families, while all other conditions occurred once in a single family only. All diagnosed families are described in Table 2, which includes two cases that were previously reported by our group: diaphanospondylodysostosis (*BMPER* gene) (Greenbaum et al., 2019) and *LMOD3*-associated Nemaline Myopathy (Berkenstadt et al., 2018). Description of US findings in families without molecular diagnosis is presented in Supplementary Table 1.

**Table 2:** Resolved 13 families- summary of main US findings, WES results and final molecular diagnosis.

Family number	Main US findings (according to fetuses)	WES type	Gene	Causative variants	Inheritance and zygosity	Diagnosis (relevant phenotype MIM number)
1	1st: Shortening of long bones (femur, humerus, tibia), IUFD 2nd: Narrow thorax, bowed femur, shortening of long bones (mostly fibula)	Quatro	*EVC2*	NM_147127.4: c.572A>T, p.Asn191Ile; NM_147127.4: c.3265C>T, p.Gln1089^∗^	AR (compound het)	Ellis-van Creveld syndrome (MIM: 225500)

2	1st: Encephalocele, large multicystic kidneys, oligohydrmanios, suspected polydactyly, lack of urinary bladder and stomach demonstration	Trio	*TCTN2*	NM_024809.4: c.1506-2A>G	AR (hom)	Meckel syndrome type 8 (MIM: 613885)
	2nd: Posterior fossa abnormality (suspected dandy-walker malformation), short and malformed corpus callosum, IUGR, SUA, small dysgenetic kidneys, urinary bladder was not visualized, oligohydramnios, hypertelorism					

3	1st: Fetal akinesia, mild polyhydramnios, small stomach, suspected right clubfoot, extended lower limbs, clenched hands, neck hyper-extension	Trio	*LMOD3*	NM_198271.4: c.723_733del, p.Asp242Glufs^∗^4; NM_198271.4: c.360dupA, p.Glu121Argfs^∗^5	AR (compound het)	Nemaline Myopathy 10 (MIM: 616165)
	2nd: Arthrogryposis, hypotonic features, abnormal posture					

4	1st: Abnormal spine and chest, unusual skull shape, echogenic cystic and horseshoe like kidneys	Single	*BMPER*	NM_133468.5: c.410T>A, p.Val137Asp	AR (hom)	Diaphanospondylodysostosis (MIM: 608022)
	2nd: Increased NT (8 mm), generalized edema, spine distortion, bilateral clubfoot, absent nasal bone					
	3rd: Reduced/lack ossification in the skull, ribs and vertebrae, protruding abdomen, short trunk					

5	1st: Distal arthrogryposis (hands), probably unilateral clubfoot, IUFD 2nd: Bilateral clubfoot	Quatro	*FKBP14*	NM_017946.3: c.568_570del, p.Lys190del	AR (hom)	Ehlers-Danlos syndrome, kyphoscoliotic type, 2 (MIM: 614557)

6	1st: Posterior urethral valve, cystic finding in kidney, suspected omphalocele 2nd: Increased NT (10 mm), cystic lesion near umbilical cord insertion site 3rd: Septated cystic hygroma, partial vermian agenesis, ARSA, omphalocele, echogenic and multicystic kidneys 4th: Increased NT (4.5 mm), facial dysmorphism (hypoplastic nasal bridge and micrognathia), echogenic kidneys, omphalocele, postaxial polydactyly, clubfoot, complex heart malformation (VSD, double outlet right ventricle, tricuspid valve regurgitation)	Quatro	*PIGN*	NM_176787.5: c.163C>T, p.Arg55^∗^; NM_176787.5: c.2283G>C, p.Lys761Asn	AR (compound het)	Multiple congenital anomalies-hypotonia-seizures syndrome 1 (MIM: 614080)

7	1st: large polycystic kidneys, oligohydramnios, moderate bilateral ventriculomegaly	Quatro	*CPT2*	NM_001330589.1: c.1239_1240del, p.Lys414Thrfs^∗^7	AR (hom)	CPT II deficiency, lethal neonatal (MIM: 608836)
	2nd: Polycystic kidneys, hydrocephalus, mega cysterna magna, macrocephaly					
	3rd: Enlarged echogenic kidneys, severe oligohydramnios, hydrocephalus, mega cysterna magna, thin corpus callosum					

8	1st: Occipital encephalocele, ventriculomegaly, mild to moderate hydronephrosis 2nd: Occipital encephalocele, ventriculomegaly, microphthalmia, cataract	Trio	*B3GALNT2*	NM_001277155.2: c.236-1G>C	AR (hom)	Muscular dystrophy-dystroglycanopathy (congenital with brain and eye anomalies, type A, 11) (MIM: 615181)

9	Short corpus callosum, suspected unilateral cataract and coloboma, IUGR (^∗^)	Single	*MED12*	NM_005120.3; c.6388C>T, p.Gln2130^∗^	XL hemizygous (maternally inherited)	Opitz-Kaveggia syndrome (MIM: 305450); Ohdo syndrome, X-linked (MIM: 300895)

10	Preaxial polydactyly of foot, syndactyly of hands (^∗∗^)	Trio	*GLI3*	NM_000168.6: c.1445G>A, p.Cys482Tyr	AD het (maternally inherited)	Greig cephalosyndactyly (MIM: 175700); Polydactyly, preaxial, type IV (MIM: 174700)

11	Shortening and bowing of long bones, poor bone mineralization, reduced skull ossification, small/narrow thorax	Trio	*COL1A2*	NM_000089.3: c.1829G>T, p.Gly610Val	*de novo* het	Osteogenesis imperfecta type 2-3 (MIM: 166210, 259420)

12	Poor ossification of skull, tibial bowing, fractures of femur, shortening of long bones	Trio	*COL1A2*	NM_000089.3: c.2260G>T, p.Gly754Cys	*de novo* het	Osteogenesis imperfecta type 2-3 (MIM: 166210, 259420)

13	Increased NT (5.7 mm), bilateral mild hypoplastic 5th finger, bilateral pyelectasis/hydronephrosis, short and thick corpus callosum, bulbous nose, mild polyhydramnios	Trio	*TCF4*	NM_001243226.2: c.2032C>T, p.Arg678^∗^	*de novo* het	Pitt-Hopkins syndrome (MIM: 610954)

*All fetuses had abnormal US findings. Cases 1–8 refer to families with two or more affected fetuses, 9–10 to families with single fetus and relevant family medical history (affected parent) and 11–13 to families with single fetus and lack of relevant family history. Cases 3 and 4 were previously reported by Berkenstadt et al. (2018) and Greenbaum et al. (2019), respectively.*

*^∗^Mother – Prominent facial dysmorphism (synophrys, small mouth and ears, mid-facial hypoplasia, prominent philtrum, dental crowding), head circumference in 10th percentile, hirsutism, menstural abnormalities and Intact cognition. She is probably a manifesting carrier. The fetus was male.*

*^∗∗^Mother – syndactyly (hands), hypotonia at infancy.*

*Abbreviations: AD, autosomal dominant; AR, autosomal recessive; ARSA, aberrant right subclavian artery; het, heterozygous; hom, homozygous. IUFD, intrauterine fetal death; IUGR, intrauterine growth restriction; MIM, Mendelian inheritance in man; NT, nuchal translucency, SUA, single umbilical artery; VSD, ventricular septal defect; XL, X-linked.*

In none of the four families without prenatal abnormal US findings was a molecular diagnosis reached. Among the 18 families with prenatal US abnormalities and positive relevant family history, a molecular diagnosis was found in 10 (55.6%). This was significantly higher compared to the 23 families with abnormal prenatal US findings and lack of relevant family history, in which the molecular cause was detected in only 3 (13%) (χ^2^(1) = 8.43, *p* = 0.004). There was no significant difference in maternal age between the two groups.

In a sub-analysis, the diagnostic rate in families with two or more fetuses with abnormal prenatal US findings was 8 out of 15 (53.3%), significantly higher than in families with prenatal US abnormalities in a mere single fetus, regardless of a relevant family history (5 out of 26, 19.2%; *p* = 0.038, Fisher’s exact test).

Looking at specific organ/body system involvement, higher diagnostic rates (more than the overall rate) were observed among families with fetal US abnormalities in the brain (60%), renal (42.9%) and musculoskeletal system (55.6%), compared to cardiac US abnormalities (16.7%) or increased NT/edema/hydrops signs (27.3%) (Table 3).

**Table 3:** Referral indications and WES yield, according to US findings in specific organ or body systems (among 41 families with US abnormalities).

Organ/body system	Number of families referred due to indication (N,%)	Families with molecular diagnosis (N,%)
Brain	10 (24.4%)	6/10 (60%)
Renal	14 (34.1%)	6/14(42.9%)
Musculoskeletal (^∗^)	18 (43.9%)	10/18 (55.6%)
Cardiac malformation	6 (14.6%)	1/6 (16.7%)
Increased NT(^∗∗^)/edema/hydrops signs	11 (26.8%)	3/11 (27.3%)

*Part of the families were classified to more than single organ/body system group, while others to none.*

*^∗^Distal limbs anomalies (e.g., polydactyly, syndactyly, clubfoot etc.) were included in this group, but not orofacial malformations.*

*^∗∗^Increased NT was defined as above 3 mm.*

Among ongoing pregnancies with US abnormalities, molecular diagnosis was found in 2 of 18 families (11.1%), both with positive relevant family history (the pregnancies were carried on). Interestingly, among 41 families with US abnormalities, significantly more families without relevant history performed WES during ongoing pregnancy (14/23, 60.9%), compared to families with positive history (4/18, 22.2%) (χ^2^(1) = 6.12, *p* = 0.013).

For all families with molecular diagnosis, options for future family planning were discussed, including prenatal diagnosis by amniocentesis or pregestational diagnosis (PGD) before next pregnancy (in autosomal dominant, recessive or X-linked inheritance) and amniocentesis to rule out recurrence due to gonadal mosaicisim (for *de novo* mutations).

### Representative Clinical Cases

Illustrative cases of two families with molecular diagnosis are presented below (numbered according to Table 2), emphasizing the challenges in the genetic counseling and the necessity of accurate US phenotyping. In the first family, with two affected fetuses (suspected recessive inheritance model), the list of potential disease causing genes in the differential diagnosis was long, and WES shortened the time of investigation. In the second family, without relevant family history, no specific diagnosis was suspected by the clinicians, and WES was probably the most cost-effective method for genetic work-up.

#### Family 2

A healthy couple of Jewish Ethiopian origin was referred for genetic counseling at 33 weeks and 2 days of gestation in their second pregnancy. A previous pregnancy was terminated at week 25, due to abnormal US findings, including encephalocele, large multicystic kidneys, oligohydramnios, suspected polydactyly and lack of urinary bladder and stomach demonstration. In the second pregnancy, NT was normal. US at early stage of pregnancy showed brain and renal malformations, but the couple did not continue follow up. At 33 week of gestation, US demonstrated posterior fossa abnormality (suspected dandy-walker malformation), a short and malformed corpus callosum, intrauterine growth retardation (IUGR), single umbilical artery (SUA), small dysgenetic kidneys, hypertelorism, and oligohydramnios. Urinary bladder was visualized. In follow up fetal brain MRI, a large cystic finding in posterior fossa was noticed, in addition to ventriculomegaly, heterotopic foci at the ventricular wall and short corpus callosum. From this deceased fetus, DNA was extracted, CMA was normal, and the couple sent a trio WES few months later. The combination of brain malformations, polydactcyly and kidneys abnormalities suggested diagnosis of Meckle–Gruber syndrome. However, since many genes are related to this disorder, and to exclude other potential disorders which were on the differential diagnosis, WES was regarded as optimal diagnostic approach. A homozygous NM_024809.4: c.1506-2A>G variant in the *TCTN2* gene (intron 13) was found in the fetus. This variant is predicted to disrupt a highly conserved acceptor splice site, and was previously described as disease causing for Meckel–Gruber type 8 syndrome in consanguineous Arab family (Shaheen et al., 2011). Other mutations in *TCTN2* were found to cause Joubert syndrome (Huppke et al., 2015). Both parents were heterozygous for this variant. The couple was advised to consider PGD in following pregnancies.

#### Family 13

A Healthy Jewish couple, both 31 years old (father from Iraq origin and mother of Ashkenazi origin) was consulted at 31 weeks (w)+5 days (d) of gestation of their first pregnancy. NT was 2.9 mm. On 16w+3d of gestation, US demonstrated elevated nuchal fold (5.7 mm), bilateral mild hypoplastic 5th finger and bilateral pyelectasis. Fetal echocardiogram was normal. Amniocentesis performed and CMA was normal. At 22w+3d, relative short and thick corpus callosum was noticed, as well as nuchal fold of 5.1–5.5 mm and mild pyelectasis. Further multiple US were carried by several experts, indicating short corpus callosum, hydronephrosis (10–13 mm), bulbous nose and mild polyhydramnios. Fetal brain MRI (at 30 weeks of gestation) demonstrated dysmorphic and short corpus callosum (<3rd percentile). The couple decided to terminate the pregnancy. In post mortem observation, facial dysmorphism, including coarseness and a bulbous nose, was noticed. Trio WES revealed a *de novo* heterozygous variant in *TCF4* gene (NM_001243226.2: c.2032C>T, p.Arg678^∗^) in the fetus, consistent with the diagnosis of Pitt-Hopkins syndrome. The fetus facial dysmorphism and the short corpus callosum supported the diagnosis. This variant creates a premature stop codon, and was previously reported in this disorder (Maduro et al., 2016). In a following pregnancy, amniocentesis was performed to exclude mutation in the fetus, due to option of germline mosaicism.

## Discussion

In this retrospective study, representing a clinical experience and practice within a tertiary referral hospital in Israel, we investigated the yield of fetal WES, from terminated and ongoing pregnancies.

The overall yield of molecular diagnosis was 28.9% (13 out of 45 families), and in families with US abnormalities, the yield was 31.7%. We noticed a significantly higher diagnostic rate in families with abnormal prenatal US findings and positive relevant family history (mainly an affected fetus from previous pregnancies), compared to families without a relevant history. This shows the importance of fetal WES indication for pregnancies with US abnormalities and positive family history, suggesting an underlining genetic disease.

Our diagnostic rate is within the range of 21–32%, seen in several studies of diagnostic yield for exome sequencing performed on fetal samples (Drury et al., 2015; Fu et al., 2018; Normand et al., 2018; Daum et al., 2019), and close to a diagnostic rate of 36.7% from exome sequencing of 278 infants in an intensive care unit (Meng et al., 2017). However, it is substantially higher than 8.5% and 10.3% found in two recent large scale fetal WES studies (Lord et al., 2019; Petrovski et al., 2019). This gap may be explained by enrichment of cases with positive family history in our cohort, and indeed our diagnostic rate in families without such history was only 13%. Interestingly, a study of 146 fetal exomes (Normand et al., 2018) has not found difference in diagnostic rate between sporadic cases and cases with significant family history. Therefore, the effect of medical family history on fetal WES yield should be further studied.

In addition, WES yield was in the range of 42.9–60% among families with involvement of brain, renal or musculoskeletal US abnormalities (higher than the overall diagnostic rate), and lower among families with cardiac malformations or increased NT/edema/hydrops signs (16.7 and 27.3%, respectively). However, since our sample size is relatively small, any interpretation of this sub-analysis should be cautious.

The resolved cases resulted in identification of causative variants in 12 genes. Some of the diagnoses are relatively common in the setting of prenatal diagnosis, such as osteogenesis imperfecta, while others are rare and include novel variants neither previously reported nor found in the databases. The latter are challenging to interpretate, due to the uncertain impact of the variants. As expected in the clinical setting, due to time and budget restrictions, no functional analysis at the molecular level was available for our cases. Thus, segregation of the variant in other family members was crucial to support diagnosis. In light of these limitations, we cannot definitely exclude that some of the molecular diagnoses are false positive. Two illustrative cases, families with molecular diagnosis of Meckel–Gruber type 8 and Pitt-Hopkins syndromes, demonstrate the clinical and genetic work-up during pregnancy and after termination, the utility of fetal WES, and its contribution for future family planning.

Well-described in the postnatal setting, prenatal WES also harbors special challenges, including numerous ethical issues which should be contemplated and addressed (Hillman et al., 2015; Jelin and Vora, 2018). The potential identification of secondary findings (also designated as incidental) and/or VUS, may have profound effect when found in ongoing pregnancies, either on parental decision making with regard to termination of pregnancy, or on the parental psychological burden following the birth of an affected child (Hillman et al., 2015; van den Veyver and Eng, 2015). One suggested strategy to circumvent some of these concerns in the prenatal setting is targeted exome testing, in which a premeditated and limited list of genes is analyzed (Pangalos et al., 2016; Chandler et al., 2018). On the other hand, according to the ACMG guidelines, no incidental findings in its selected list of actionable genes should be reported in prenatal samples.

Other concerns include issues related to turnaround time, lack of full coverage of potentially relevant genes, and inaccurate and limited phenotyping capabilities for fetuses, resulting in insufficient data for the WES interpretation (Aarabi et al., 2018; Best et al., 2018). Postnatal clinical evaluations or autopsies may be of great value, improving the diagnostic accuracy and yield, with new data not observed in US examination (Aarabi et al., 2018).

Our study is based on a cohort of consecutive (non-selected) series of families for which WES was performed in certified clinical laboratories, on a clinical base (not for research purpose as the main goal), and fully paid for, usually by families. In this regard, the study represents a real-world situation in prenatal counseling practicality, where the diagnosis is pursued due to a clinical need, and not within a research setup. Of note, most families with US abnormalities and lack of relevant family history asked to perform WES during ongoing pregnancy (14/23, 60.9%), while most families with positive history sent it after TOP (14/18, 77.8%). Four families asked to perform fetal WES during ongoing pregnancy even when no US abnormalities were found, mainly for detection of *de novo* pathogenic variants or recessive disorders. Three of the four families had offsprings affected by a severe single-gene condition. In these cases, WES was sent even when the specific pathogenic variant in the family was excluded in the fetus prior to WES submission (by Sanger sequencing), since families asked to search for other potential genetic disorders in the fetus.

The current study population is relatively homogenous in terms of demographic characteristics, composed of 45 families from Jewish ancestry, without consanguinity (except one family), all living in Israel and followed by a single prenatal genetic clinic. For most families in our cohort, genetic evaluation in addition to WES included CMA as well as parental screening for common genetic diseases in the Jewish population. In this aspect, the reported cohort might not be representative of other patient populations. For example, it is plausible that diagnostic yield may be even higher in WES pursued for fetuses of consanguineous couples.

The majority of families with fetal abnormal US findings seen in our clinic (several hundred each year), decided not to perform fetal WES, due to multiple considerations, including financial issues. Therefore, our study is biased toward families with high motivation to reach molecular diagnosis and available financial resources. An additional limitation of the present study is that WES was sent to three different clinical services. Therefore, sequencing was performed using different protocols and kits, and diverse bioinformatics pipelines were used for data analysis, potentially affecting the diagnostic yield due to inter-laboratory differences. Uniform sequencing and analysis in one laboratory, applied to all families, may have been an advantage. In addition, revision of WES data was not done for most unresolved cases. Together with our relative small sample size, the results should be considered as preliminary, and warrant further study.

To conclude, our study demonstrates that fetal WES is especially indicated when positive relevant family history is present, or in fetuses with specific types of congenital malformation. Following careful patient selection, clinical fetal WES is an important tool for evaluating fetuses with abnormal US findings, with important implications for both ongoing and future pregnancies.

## Ethics Statement

The study was carried out in accordance with the recommendations of the Institutional Review Board (IRB) committee of SMC, and was approved by it.

## Author Contributions

LG and MB designed the study, provided genetic counseling to families, researched the data, and wrote the manuscript. BP-S and EP contributed to study design and interpretations of data. SE-B, MD-K, AB-Z, NG, HP, MR, and AR-R provided genetic counseling to families, collected the data, and revised the manuscript. AR assisted in statistical analysis. HR-W, AB-A, PB, and OB participated in bioinformatics analysis and revised the manuscript.

## Conflict of Interest Statement

AB-A and PB are employees of Centogene AG. The remaining authors declare that the research was conducted in the absence of any commercial or financial relationships that could be construed as a potential conflict of interest.

## Footnotes

1 https://www.centogene.com/

2 http://www.hgmd.cf.ac.uk/

3 https://www.ncbi.nlm.nih.gov/clinvar/

4 https://www.centomd.com/

5 http://exac.broadinstitute.org/

6 https://www.cegat.de/
