# Supplementary material for: Evaluation of Diagnostic Yield in Fetal Whole-Exome Sequencing: A Report on 45 Consecutive Families
Source: Front Genet. 2019 Jun 25;10:425. doi: 10.3389/fgene.2019.00425 (PMC6688107; doi:10.3389/fgene.2019.00425)
Supplement: Supplementary file 1 [file Table_1.doc]

**Greenbaum L. et al., Evaluation of Diagnostic Yield in Fetal Whole-Exome Sequencing: A Report on 45 Consecutive Families**

**Supplementary Table 1: Summary of all families without molecular diagnosis**

| **Family Number** | **Main US findings (according to fetuses)** | **WES type** |
| --- | --- | --- |
| 1 | 1st: Congenital heart defect (VSD+TGA) | Trio |
|  | 2nd: Pleural effusion, diaphragmatic eventration |  |
| 2 | 1st: Hypoplastic left heart, aortic atresia, PRUV | Quatro |
|  | 2nd: Increased NT (4mm), AV canal, cleft lip and palate, horseshoe kidneys |  |
| 3 | 1st: Unilateral cleft lip and palate, unilateral clubfoot (*) | Trio |
|  | 2nd: Bilateral cleft lip (potentially cleft palate) |  |
| 4 | 1st: Shortening of long bones (5th percentile), polyhydramnios, facial dysmorphism (low-set and small ears), echogenic kidneys | Trio |
|  | 2nd: Unilateral club foot, progressive shortening of long bones (3rd percentile), abnormal posture of right hand |  |
| 5 | 1st: Moderate ventriculomegaly (week 16 of gestation) | Quatro |
|  | 2nd: Moderate ventriculomegaly (week 16 of gestation) |  |
| 6 | 1st pregnancy: Hypoplastic left heart | Quatro |
|  | 2nd pregnancy: Hypoplastic right heart, pulmonary atresia, severe tricuspid valve regurgitation |  |
| 7 | 1st pregnancy: Bilateral cleft lip and palate | Trio |
|  | 2nd pregnancy: Unilateral cleft lip |  |
| 8 | Macrocephaly (>95 percentile), moderate and asymmetric venticulomegaly (**) | Trio |
| 9 | No visualization of gall bladder, preaxial polydactyly, bifid thumb, left persistent superior vena cava, mesocardia | Trio |
| 10 | Severe hydrops, ascites, hydrothorax | Trio |
| 11 | Increased NT, posterior neck edema, two wormian bones in posterior fontanella and mildly malformed skull | Trio |
| 12 | Moderate ventriculomegaly, periventricular hyperechogeneity, wide corpus callosum | Trio |
| 13 | IUGR, transient pleural effusion, mild echogenic bowel | Trio |
| 14 | No visualization of gallbladder, low conus medularis, minor abnormality of portal veins | Trio |
| 15 | Unilateral postaxial polydactyly (foot) | Trio |
| 16 | Bilateral clubfoot, SUA, vertebral malformation, sacral agenesis | Trio |
| 17 | Bilateral small kidneys (3rd percentile, in multiple scans) with normal structure | Trio |
| 18 | Cystic hygroma (7 mm), bilateral pleural effusion, SUA, VSD | Trio |
| 19 | Unilateral multicystic dysplastic kidney, presacral cyst (suspected Hematocolpus or anal atresia) | Trio |
| 20 | Echogenic cavitated cyst (suspected CPAM or CCAM) | Trio |
| 21 | Increased NT (3.1 mm) | Trio |
| 22 | Bilateral multicystic and enlarged kidneys | Trio |
| 23 | Significant polyhydramnios, enlarged kidney with solid cystic mass (unilateral) , enlarged pancreas, hepatomegaly | Trio |
| 24 | Partial agenesis of corpus callosum, small cerebellum, horseshoe kidney | Trio |
| 25 | Polyhydramnios, bilateral adducted thumbs | Trio |
| 26 | Neck edema, mild pleural effusion and ascites | Trio |
| 27 | IUGR (suspected), mild unilateral, echogenic kidney | Single |
| 28 | IUGR (5-10 percentile), relative shortening of long bones (3-5 percentile) | Trio |
| 29 | No abnormal findings (***) | Trio |
| 30 | No abnormal findings | Trio |
| 31 | No abnormal findings | Single |
| 32 | No abnormal findings | Single |

Cases 1-7 refer to families with two affected fetuses, 8 to a family with single fetus and a relevant family medical history (affected father), and 9-28 to families with single fetus and lack of relevant family history. In families 29-32, no abnormal US findings were noted.

* Father - Cleft lip and palate.

** Father - Macrocephaly (97th percentile).

*** Parents are first degree cousins.

Abbreviations: CCAM - congenital cystic adenomatoid malformation, CPAM - congenital pulmonary airway malformation, IUGR- intrauterine growth restriction, NT- nuchal translucency, PRUV- persistant right umbilical vein, SUA – single umbilical artery, TGA - transposition of great arteries, VSD- ventricular septal defect.
